# *TCF4* trinucleotide repeat expansion drives distinct proteomic signatures in Fuchs endothelial corneal dystrophy

**DOI:** 10.1038/s41598-026-43789-x

**Published:** 2026-03-21

**Authors:** Taichi Yuasa, Tatsuya Nakagawa, Tetsuro Honda, Go Nishiuchi, Masakazu Sato, Ayumi Tokunaga, Makiko Nakahara, Theofilos Tourtas, Ursula Schlötzer-Schrehardt, Friedrich Kruse, Prema Padmanabhan, Amit Chatterjee, Gajanan Sathe, Vivek Ghose, Narayanan Janakiraman, Noriko Koizumi, Sailaja V. Elchuri, Naoki Okumura

**Affiliations:** 1 Department of Biomedical Engineering, Faculty of Life and Medical Sciences, Doshisha University, Kyotanabe, 610-0394 Japan; 2 Department of Ophthalmology, University of Erlangen-Nürnberg, Erlangen, Germany; 3 Department of Cornea and Refractive Surgery, Sankara Nethralaya, Chennai, India; 4 Department of Nanobiotechnology, Vision Research Foundation, Sankara Nethralaya, 18 College Road, Chennai, Tamil Nadu 600 006 India; 5 Institute of Bioinformatics, Bangalore, India; 6 Manipal Academy of Higher Education, Manipal, India

**Keywords:** Eye diseases, Experimental models of disease

## Abstract

The aims of this study were to use an isogenic cell model system to investigate the proteomic consequences of *TCF4* trinucleotide repeat expansion in Fuchs endothelial corneal dystrophy (FECD) and to identify potential molecular pathways contributing to disease pathogenesis. We used our previously established immortalized FECD cells (iFECD) that harbor CTG repeat expansion in *TCF4* and CRISPR/Cas9 genome editing to generate an isogenic counterpart (iFECD *TCF4*ΔCTG) in which the expansion was deleted. Comprehensive proteomic analysis was then performed using tandem mass tag (TMT)-labeled quantitative LC-MS/MS. The resulting data were subjected to differential expression analysis, functional enrichment analysis, and protein-protein interaction network construction to elucidate the molecular impact of the CTG repeat expansion. Deletion of the CTG repeat expansion significantly altered the corneal endothelial proteome, with 90 upregulated and 111 downregulated proteins (|log_2_ fold change| ≥ 0.5, *P*-value < 0.05). Functional enrichment analysis revealed that the downregulated proteins were predominantly associated with extracellular matrix organization and cell–substrate adhesion pathways, while the upregulated proteins were enriched in interferon signaling and antigen-processing pathways. The most significantly upregulated proteins included neuropilin-1, Cip1-interacting zinc finger protein, and protein-glutamine gamma-glutamyltransferase 2, while protein phosphatase 1 regulatory subunit 14 C, alpha-crystallin B chain, and 14-3-3 protein sigma showed the greatest downregulation. Our findings demonstrate that *TCF4* CTG repeat expansion significantly impacts the corneal endothelial proteome, particularly affecting the extracellular matrix and cell adhesion proteins that likely contribute to guttae formation. These proteomic alterations provide mechanistic insights connecting trinucleotide repeat expansion to endothelial dysfunction and suggest potential therapeutic targets for FECD.

**Supplementary Information:**

The online version contains supplementary material available at 10.1038/s41598-026-43789-x.

## Introduction

Fuchs endothelial corneal dystrophy (FECD) is the most common corneal dystrophy and a leading cause of corneal transplantation worldwide. Clinically, FECD is characterized by guttae formation on Descemet’s membrane. The guttae cause light scatter and progressive corneal endothelial cell loss, resulting in corneal edema and significant impairment of visual function^1–3^. While several genetic factors have been implicated in FECD pathogenesis^4–6^, a breakthrough discovery came about in 2012 when Wieben et al. identified a striking association between FECD and an expansion of CTG trinucleotide repeats within intron 3 of the *TCF4* gene^7^. Subsequent studies have confirmed that approximately 70% of FECD patients harbor this repeat expansion, making it the most prevalent genetic risk factor for the disease, particularly in Caucasian populations^8–13^.

Despite the substantial evidence now supporting FECD as a trinucleotide repeat expansion disorder, the molecular mechanisms connecting the *TCF4* CTG expansion to corneal endothelial dysfunction remain incompletely understood^2^. Current models have proposed several pathogenic pathways, including RNA toxicity through sequestration of splicing factors, repeat-associated non-AUG (RAN) translation, and altered *TCF4* expression^11,14–18^. Transcriptomic analyses have identified widespread alterations in gene expression and splicing patterns in the FECD corneal endothelium^16,19^; however, due to post-transcriptional regulation and other factors, the observed gene expression changes do not always correlate directly with protein abundance^20,21^. Furthermore, whether these mRNA-level alterations translate to functional proteomic changes remains largely unexplored^20,21^.

Proteomic investigation of FECD has been limited by technical challenges in obtaining sufficient tissue from the single-cell layer of the corneal endothelium. This knowledge gap is particularly significant since the hallmark features of FECD are fundamentally linked to protein-level changes in extracellular matrix composition and cellular function^22^. Therefore, in the present study, we leveraged our previously established immortalized FECD cell line harboring the *TCF4* CTG repeat expansion to create an isogenic experimental system using CRISPR/Cas9 genome editing. We generated a matched cell line (iFECD *TCF4*ΔCTG) in which the trinucleotide repeat expansion was precisely deleted while preserving all other genetic features. Using quantitative proteomic analysis, we identified differentially expressed proteins and characterized the pathways affected by the repeat expansion, thereby directly addressing how *TCF4* trinucleotide repeat expansion influences protein expression in FECD.

## Results

### Deletion of the CTG repeat expansion in iFECD cells

**Fig. 1:**
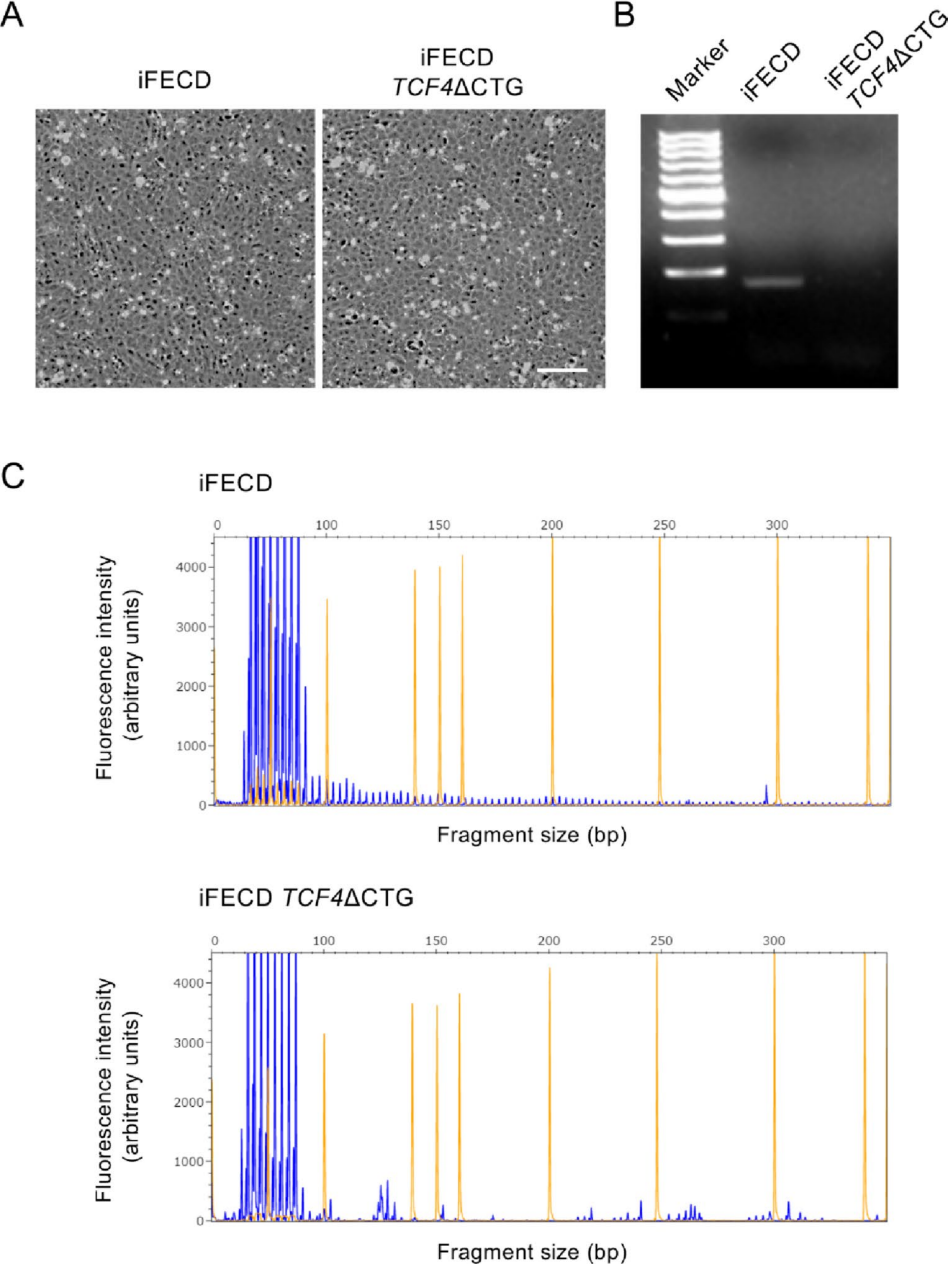
Generation and characterization of the *TCF4* CTG repeat expansion knockout cell line. ( **A**) Representative phase contrast micrographs of immortalized Fuchs endothelial corneal dystrophy (FECD) cells (iFECD) and their isogenic counterpart following CRISPR/Cas9-mediated deletion of the *TCF4* CTG repeat expansion (iFECD *TCF4*ΔCTG). Both cell lines exhibit typical corneal endothelial morphology, with polygonal cells forming intact monolayers, indicating that deletion of the trinucleotide repeat expansion did not alter cellular morphology. The scale bar represents 50 μm. (**B**) Representative results from three independent experiments showing PCR analysis of the *TCF4* trinucleotide repeat region. The expanded allele is clearly visible in parental iFECD cells but absent in iFECD *TCF4*ΔCTG cells, confirming successful deletion of the CTG repeat expansion. ( **C**) Representative triplet repeat primed PCR (TP-PCR) electropherograms from three biological replicates. Triplet repeat primed PCR (TP-PCR) electropherograms. The iFECD sample (top) displays the characteristic “saw-tooth” pattern extending beyond 40 repeats, indicating the presence of a pathogenic expansion. In contrast, the iFECD *TCF4*ΔCTG sample (bottom) shows only normal-length alleles (< 40 repeats), demonstrating complete elimination of the expanded allele.

Phase contrast microscopy revealed that both iFECD and CRISPR/Cas9-edited iFECD *TCF4*ΔCTG cells maintained a typical corneal endothelial morphology, exhibiting a polygonal cell shape and forming intact monolayers (Fig. 1A). These observations were consistently obtained from three independent biological replicates, and representative images are shown. Deletion of the CTG repeat expansion did not induce any observable morphological changes or cytotoxicity. PCR analysis of the *TCF4* trinucleotide repeat region demonstrated successful genomic editing, with the expanded allele clearly visible in the parental iFECD cells but absent in the iFECD *TCF4*ΔCTG cells (Fig. 1B). The full, unprocessed agarose gel image corresponding to this PCR analysis is provided in Supplementary Fig. 1. Further characterization using triplet repeat primed PCR (TP-PCR) confirmed that while iFECD cells harbored CTG repeat expansions exceeding 40 repeats, as evidenced by the characteristic “saw-tooth” pattern in the electropherogram, the iFECD *TCF4*ΔCTG cells showed a complete elimination of these expanded alleles (Fig. 1C). Representative data from three independent experiments are presented. These results confirmed the successful generation of an isogenic cell line lacking the CTG repeat expansion while maintaining normal cellular morphology.

### Proteomic analysis reveals distinct expression patterns following deletion of the ***TCF4*** CTG repeat

**Fig. 2:**
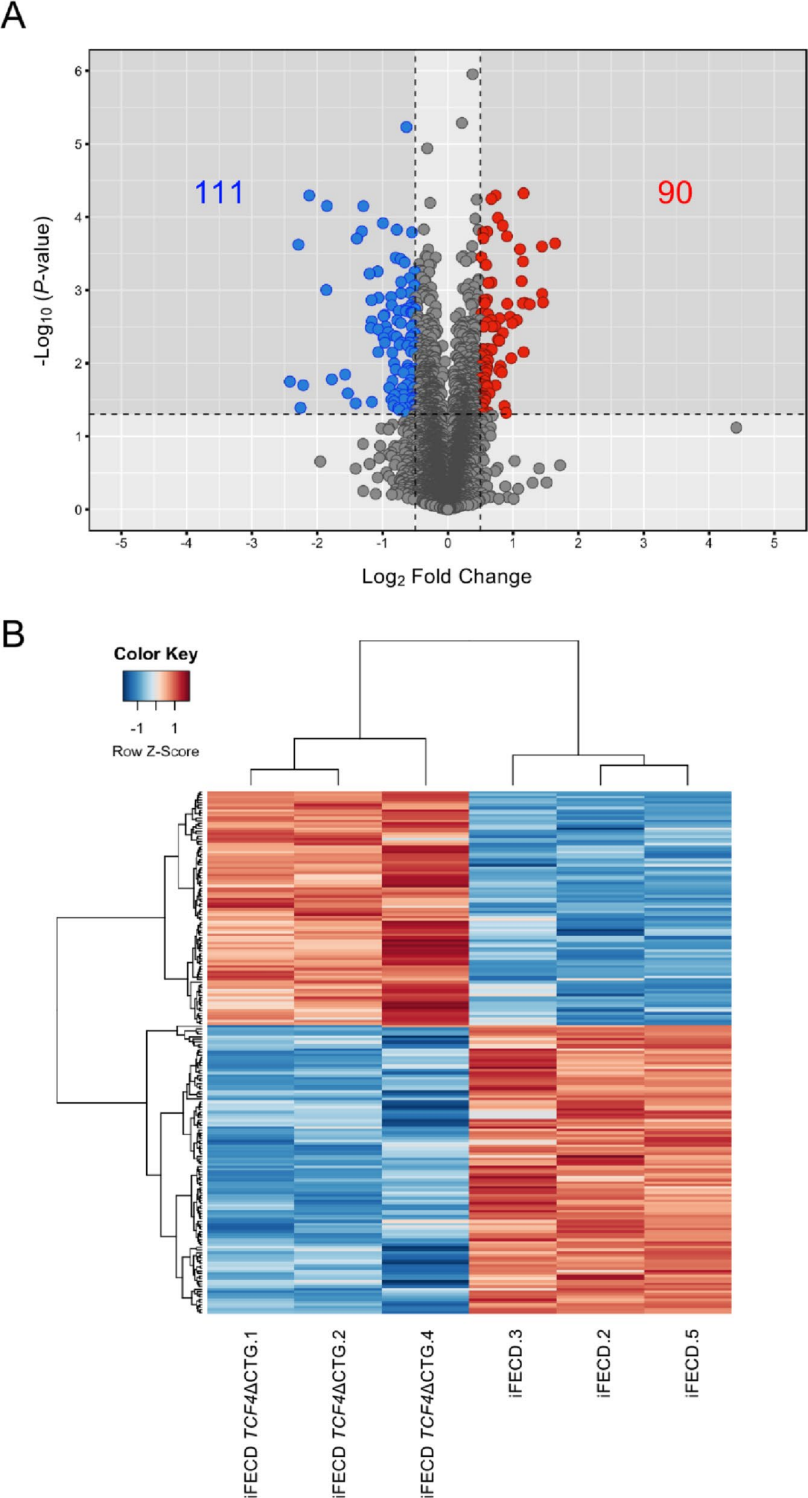
Proteomic analysis reveals distinct expression profiles following *TCF4* CTG repeat deletion. (**A**) Volcano plot illustrating differentially expressed proteins between parental iFECD cells and iFECD *TCF4*ΔCTG cells identified by LC-MS/MS analysis. Proteomic profiling was performed using three independent biological replicates for each cell line. Red circles (*n* = 90) represent significantly upregulated proteins, while blue circles (*n* = 111) indicate significantly downregulated proteins in iFECD *TCF4*ΔCTG compared to iFECD cells (criteria: |log_2_ fold change| ≥ 0.5, *P*-value < 0.05). The x-axis represents log_2_ fold change (iFECD *TCF4*ΔCTG/iFECD), and the y-axis shows statistical significance as -log_10_ (*P*-value). (**B**) Hierarchical clustering heatmap of differentially expressed proteins generated using the heatmap.2 function in R. Each group (iFECD and iFECD *TCF4*ΔCTG) included three independent biological replicates, and the heatmap demonstrates clear separation between iFECD samples (right columns) and iFECD *TCF4*ΔCTG samples (left columns). Protein expression levels are normalized to Z-scores, with red indicating relatively higher expression and blue indicating relatively lower expression. Distinct clustering patterns based on triplicate samples confirm reproducible proteomic changes associated with CTG repeat deletion.

We investigated the molecular consequences of the *TCF4* trinucleotide repeat expansion by performing a comprehensive proteomic analysis comparing parental iFECD cells with the CRISPR-edited iFECD *TCF4*ΔCTG cells. Proteomic profiling was conducted using three independent biological replicates for each cell line. Quantitative mass spectrometry identified significant alterations in the protein expression patterns between these isogenic cell lines. A volcano plot visualization of the proteomic data revealed 201 differentially expressed proteins (DEPs) meeting our statistical criteria (|log_2_ fold change| ≥ 0.5, *P*-value < 0.05), with 90 proteins significantly upregulated (red dots) and 111 proteins significantly downregulated (blue dots) by the CTG repeat deletion (Fig. 2A).

Hierarchical clustering analysis of these expression profiles demonstrated a clear separation between the iFECD and iFECD *TCF4*ΔCTG groups, based on triplicate biological samples, confirming the reproducibility of the proteomic changes across biological replicates (Fig. 2B). The heat map representation in Fig. 2B displays normalized Z-scores for each protein, with red indicating higher expression and blue indicating lower expression. This distinct clustering pattern underscores the substantial impact of the CTG repeat expansion on the corneal endothelial cell proteome.

Tables 1 and 2 list the top 30 most significantly upregulated and downregulated proteins in the iFECD *TCF4*ΔCTG cells compared to the parental iFECD cells. The three most upregulated proteins were neuropilin-1 (log_2_ fold change = 1.64), Cip1-interacting zinc finger protein (log_2_ fold change = 1.46), and protein-glutamine gamma-glutamyltransferase 2 (log_2_ fold change = 1.44). Conversely, the three most downregulated proteins were protein phosphatase 1 regulatory subunit 14 C (log_2_ fold change = − 2.42), alpha-crystallin B chain (log_2_ fold change = − 2.29), and 14-3-3 protein sigma (log_2_ fold change = − 2.26). These DEPs represent potential downstream effectors of the *TCF4* CTG repeat-mediated pathology in FECD.

**Table 1:** Top 30 upregulated proteins in FECD corneal endothelial cells following *TCF4* CTG repeat expansion deletion.

Protein name	Refseq protein ID	Log_2_ (fold change)	*P*-value
Neuropilin-1 isoform X1	XP_006717584.1	1.64	2.29 × 10^− 4^
Cip1-interacting zinc finger protein isoform 5	NP_001244904.1	1.46	1.47 × 10^− 3^
Protein-glutamine gamma-glutamyltransferase 2 isoform X1	XP_011527330.1	1.44	1.12 × 10^− 3^
Paraneoplastic antigen Ma2 isoform X1	XP_011542667.1	1.44	2.54 × 10^− 4^
Syndecan-1 isoform X1	XP_005262677.1	1.25	1.57 × 10^− 3^
Voltage-dependent calcium channel subunit alpha-2/delta-1 isoform X6	XP_005250629.1	1.18	1.49 × 10^− 3^
Ferritin light chain isoform X1	XP_024307215.1	1.16	7.05 × 10^− 3^
Collagen alpha-1(XVIII) chain isoform 3 preproprotein	NP_569711.2	1.16	4.73 × 10^− 5^
BTB/POZ domain-containing protein KCTD12	NP_612453.1	1.15	4.06 × 10^− 4^
Protein YIPF5 isoform a	NP_110426.4	1.15	1.51 × 10^− 3^
Integrin alpha-2 precursor	NP_002194.2	1.13	7.54 × 10^− 4^
Isochorismatase domain-containing protein 1	NP_057132.2	1.10	2.76 × 10^− 4^
Microtubule-associated protein 2 isoform X1	XP_024308659.1	1.06	2.57 × 10^− 3^
Antigen peptide transporter 1 isoform 1	NP_000584.2	0.99	2.84 × 10^− 3^
Jupiter microtubule associated homolog 1 isoform 1	NP_057269.1	0.97	8.56 × 10^− 3^
Collagen alpha-3(VI) chain isoform 1 precursor	NP_004360.2	0.95	2.32 × 10^− 3^
Stromal cell-derived factor 2-like protein 1 precursor	NP_071327.2	0.91	1.54 × 10^− 3^
Filamin-binding LIM protein 1 isoform X2	XP_011539919.1	0.90	1.83 × 10^− 4^
Discoidin, CUB and LCCL domain-containing protein 2 precursor	NP_563615.3	0.89	4.77 × 10^− 2^
Splicing factor U2AF 35 kDa subunit-like protein isoform 1	NP_001307575.1	0.87	3.85 × 10^− 2^
Uridine phosphorylase 1 isoform X1	XP_011513814.1	0.84	3.85 × 10^− 3^
Rho-related BTB domain-containing protein 3	NP_055714.3	0.84	1.31 × 10^− 4^
2’-5’-oligoadenylate synthase 2 isoform 1	NP_058197.2	0.83	1.34 × 10^− 2^
Sterile alpha and TIR motif-containing protein 1 precursor	NP_055892.2	0.80	1.09 × 10^− 2^
COUP transcription factor 2 isoform a	NP_066285.1	0.79	2.44 × 10^− 3^
Dysferlin isoform X1	XP_005264641.1	0.79	4.90 × 10^− 3^
Cytochrome c oxidase subunit 6B1	NP_001854.1	0.78	1.24 × 10^− 2^
HLA class I histocompatibility antigen, A-1 alpha chain A*03:01:0:01 precursor	NP_002107.3	0.77	4.74 × 10^− 3^
Proteasome subunit beta type-9 precursor	NP_002791.1	0.77	1.03 × 10^− 4^
HLA class I histocompatibility antigen, A-1 alpha chain isoform X1	XP_024308303.1	0.73	5.06 × 10^− 5^

**Table 2:** Top 30 downregulated proteins in FECD corneal endothelial cells following *TCF4* CTG repeat expansion deletion.

Protein name	Refseq protein ID	Log_2_ (fold change)	*P*-value
Protein phosphatase 1 regulatory subunit 14 C	NP_112211.1	-2.42	1.79 × 10^− 2^
Alpha-crystallin B chain isoform 1	NP_001276736.1	-2.29	2.38 × 10^− 4^
14-3-3 protein sigma	NP_006133.1	-2.26	4.08 × 10^− 2^
Connector enhancer of kinase suppressor of ras 2 isoform 1	NP_055742.2	-2.22	2.00 × 10^− 2^
Fructose-1,6-bisphosphatase isozyme 2	NP_003828.2	-2.12	5.06 × 10^− 5^
Keratin, type I cytoskeletal 19	NP_002267.2	-1.86	9.95 × 10^− 4^
Protein HEATR9 isoform 1	NP_689994.2	-1.85	7.06 × 10^− 5^
Fructose-1,6-bisphosphatase 1	NP_000498.2	-1.78	1.67 × 10^− 2^
Neural cell adhesion molecule 1 isoform 5 precursor	NP_001229536.1	-1.57	1.43 × 10^− 2^
Gremlin-1 isoform X1	XP_016877566.1	-1.53	2.58 × 10^− 2^
Histone H1.0	NP_005309.1	-1.41	3.54 × 10^− 2^
Protein AHNAK2 isoform 1	NP_612429.2	-1.39	1.98 × 10^− 4^
Carbonic anhydrase 3	NP_005172.1	-1.32	1.57 × 10^− 4^
GTP cyclohydrolase 1 feedback regulatory protein	NP_005249.1	-1.29	7.09 × 10^− 5^
Tropomyosin alpha-1 chain isoform Tpm1.8cy	NP_001288218.1	-1.20	5.96 × 10^− 4^
Carbonic anhydrase 13	NP_940986.1	-1.18	3.29 × 10^− 3^
Nephronectin isoform X5	XP_011530126.1	-1.17	1.37 × 10^− 3^
Protein S100-A4	NP_062427.1	-1.17	2.66 × 10^− 3^
Interleukin-18 isoform X2	XP_011541108.1	-1.16	3.39 × 10^− 2^
Epiplakin isoform X3	XP_016869381.1	-1.07	5.56 × 10^− 4^
Syntaxin-binding protein 6 isoform 2	NP_001338869.1	-1.07	7.05 × 10^− 3^
Laminin subunit alpha-5 isoform X1	XP_006723859.1	-1.07	3.45 × 10^− 3^
Creatine kinase B-type isoform 2	NP_001349460.1	-1.06	1.28 × 10^− 3^
Myelin expression factor 2 isoform a	NP_057216.2	-0.99	1.22 × 10^− 4^
Heparanase isoform 1 preproprotein	NP_006656.2	-0.98	2.26 × 10^− 3^
Acid sphingomyelinase-like phosphodiesterase 3b isoform X1	XP_011539561.1	-0.98	4.51 × 10^− 3^
Cellular retinoic acid-binding protein 2	NP_001186652.1	-0.97	2.19 × 10^− 3^
Cingulin-like protein 1 isoform X1	XP_016878174.1	-0.97	5.23 × 10^− 3^
DNA-binding protein RFXANK isoform a	NP_003712.1	-0.95	3.16 × 10^− 3^
Cystathionine gamma-lyase isoform 1	NP_001893.2	-0.91	3.75 × 10^− 3^

### Western blotting validation of proteomics data

**Fig. 3:**
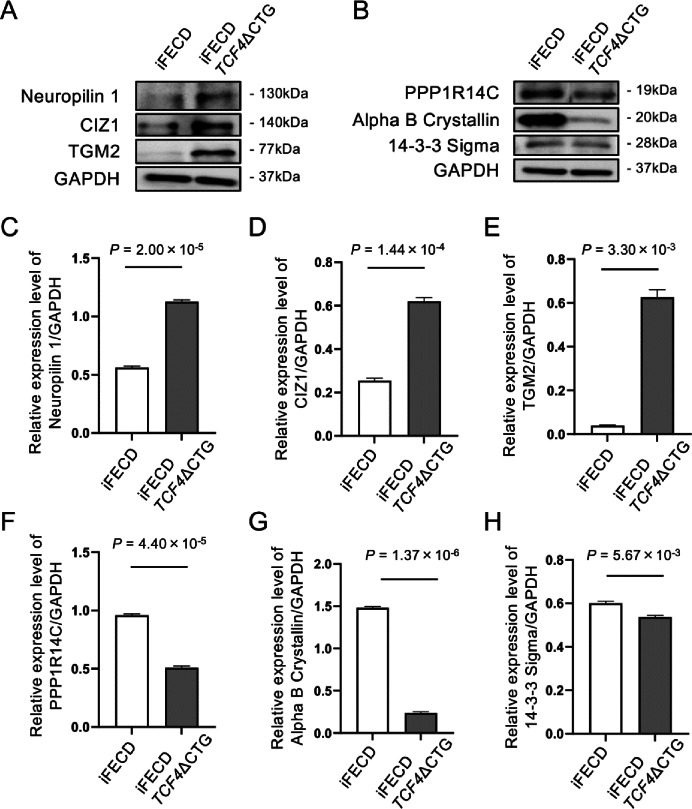
Western blotting validation of proteomics data. (**A**) Western blot analysis of the top three upregulated proteins (Neuropilin 1, CIZ1, and TGM2) in iFECD and iFECD *TCF4*ΔCTG cell lysates. (**B**) Western blot analysis of the top three downregulated proteins (PPP1R14C, Alpha B Crystallin, and 14-3-3 Sigma) in iFECD and iFECD *TCF4*ΔCTG cell lysates. GAPDH was used as a loading control. The results were consistent with the quantitative proteomics data, confirming the differential expression of these key proteins. (**C–H**) Densitometric quantification of Western blot bands for (**C**) CIZ1, (**D**) Neuropilin 1, (**E**) TGM2, (**F**) PPP1R14C, (**G**) Alpha B Crystallin, and (**H**) 14-3-3 Sigma. Relative expression levels were normalized to GAPDH. Data are presented as mean ± SEM. P-values were determined using Welch’s t-test (*n* = 3 independent experiments).

To validate the proteomic findings, we performed Western blotting for three of the most significantly upregulated proteins (Neuropilin 1, CIZ1, and TGM2) and three of the most significantly downregulated proteins (PPP1R14C, Alpha B Crystallin, and 14-3-3 sigma). The results confirmed higher expression of Neuropilin 1, CIZ1, and TGM2(Fig. 3A) and lower expression of PPP1R14C, Alpha B Crystallin, and 14-3-3 sigma in iFECD *TCF4*ΔCTG cells (Fig. 3B), consistent with the quantitative mass spectrometry data. Densitometric quantification of the Western blot bands, normalized to GAPDH, confirmed these trends. Specifically, the expression levels of Neuropilin 1, CIZ1, and TGM2 were approximately 2.01, 2.43, and 16.1 fold higher, respectively, in iFECD *TCF4*ΔCTG cells compared with iFECD controls (Fig. 3C–E). Conversely, PPP1R14C, Alpha B Crystallin, and 14-3-3 sigma were reduced to roughly 0.53, 0.16, and 0.89 fold, respectively, relative to iFECD (Fig. 3F–H). These quantitative changes robustly corroborate the proteomic dataset. The corresponding unprocessed full-length membrane images are provided in Supplementary Fig. 2.

### Functional enrichment and pathway analysis of the *TCF4* CTG repeat-associated proteome

**Fig. 4:**
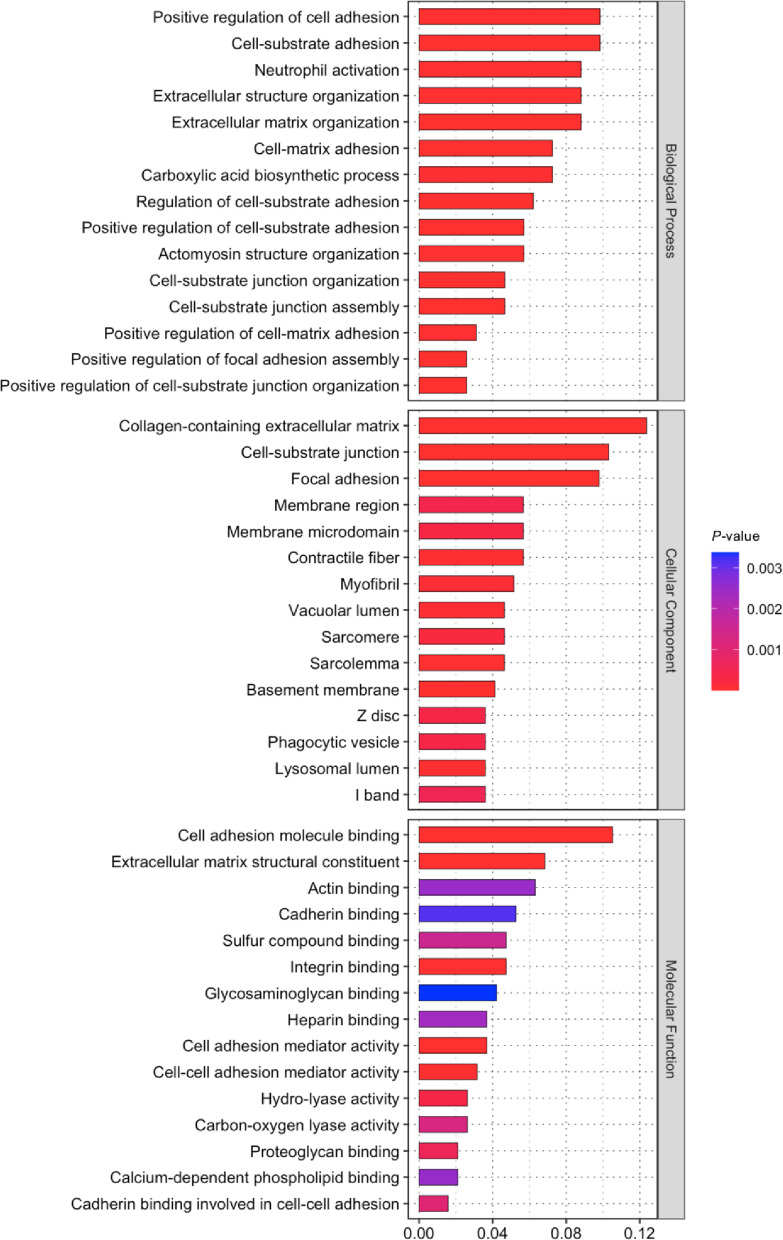
Gene ontology (GO) enrichment analysis of differentially expressed proteins. GO enrichment analysis of proteins differentially expressed between iFECD and iFECD *TCF4*ΔCTG cells was performed using ClusterProfiler. The most significantly enriched GO terms (*P*-value < 0.05) are shown for biological processes (top panel), cellular components (middle panel), and molecular functions (bottom panel). The x-axis represents the gene ratio (the proportion of DEPs in each GO term relative to all DEPs), while color intensity indicates statistical significance. Cell adhesion and extracellular matrix-related terms are prominently enriched across all three GO categories.

Gene Ontology (GO) enrichment analysis revealed distinct functional patterns affected by *TCF4* CTG repeat deletion (Fig. 4). The most significantly enriched functions were biological processes, cellular components, and molecular functions, which highlighted two major functional categories: cell adhesion and extracellular matrix (ECM) organization. The cell adhesion-related pathways were prominently represented across all GO categories and included (1) positive regulation of cell adhesion, cell-substrate adhesion, and cell-matrix adhesion in biological processes; (2) cell-substrate junction and focal adhesion in cellular components; and (3) cell adhesion molecule binding, actin binding, and integrin binding in molecular functions. Concurrently, significant enrichment was evident in ECM-related pathways, including (1) extracellular structure organization and extracellular matrix organization in biological processes; (2) collagen-containing ECM in cellular components; and (3) ECM structural constituents in molecular functions.

**Fig. 5:**
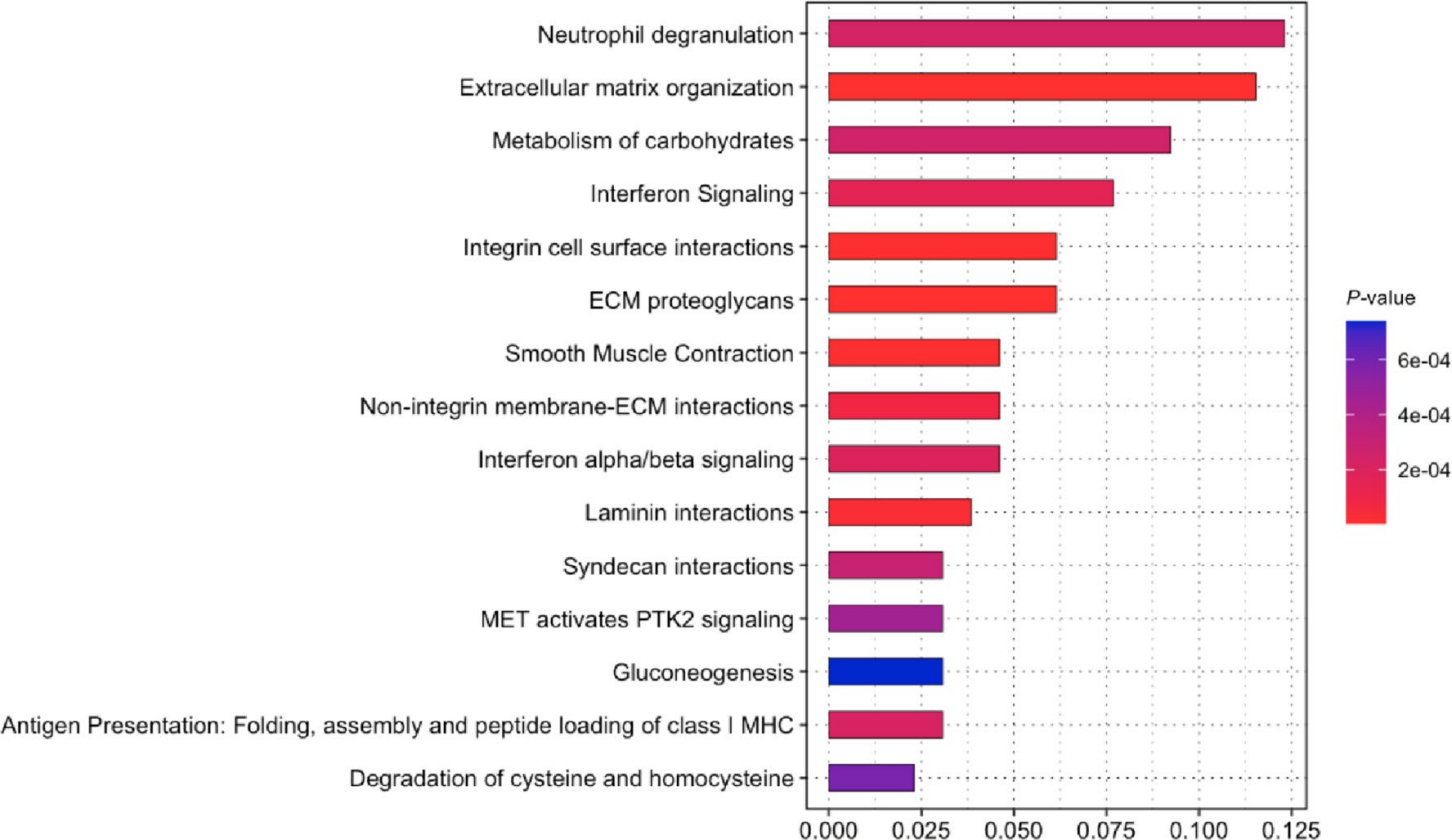
Reactome pathway enrichment analysis of differentially expressed proteins. Reactome pathway analysis of proteins differentially expressed between iFECD and iFECD *TCF4*ΔCTG cells. The top 15 significantly enriched pathways (*P*-value < 0.05) are shown, with the x-axis representing gene ratio and color intensity indicating statistical significance. Neutrophil degranulation, extracellular matrix organization, and interferon signaling pathways emerged as highly enriched functional categories, suggesting their potential role in *TCF4* repeat expansion–mediated pathology in FECD.

Complementary Reactome pathway analysis further confirmed the importance of ECM-related biological processes (Fig. 5). The most significantly enriched pathways included neutrophil degranulation, extracellular matrix organization, metabolism of carbohydrates, interferon signaling, integrin cell surface interactions, and ECM proteoglycans. Other enriched pathways included non-integrin membrane–ECM interactions, laminin interactions, and syndecan interactions.

**Fig. 6:**
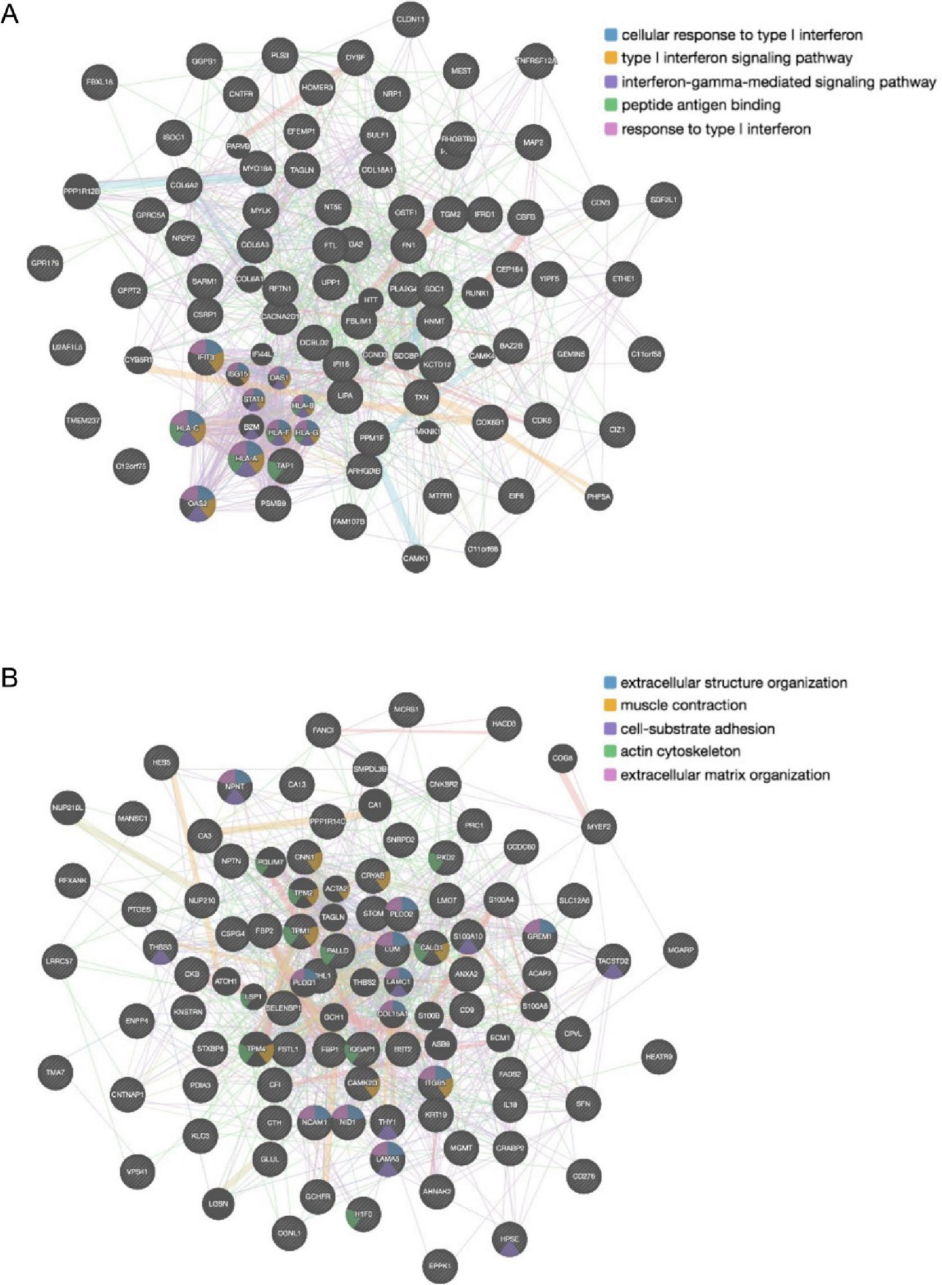
Protein–protein interaction (PPI) networks of differentially expressed proteins. (**A**) Protein–protein interaction networks constructed using GeneMANIA for proteins differentially expressed following *TCF4* CTG repeat deletion. Network of 90 upregulated proteins in iFECD *TCF4*ΔCTG cells compared to iFECD cells. Colored edges represent functional associations related to interferon signaling pathways, including cellular response to type I interferon (blue), type I interferon signaling pathway (orange), interferon-gamma-mediated signaling pathway (green), peptide antigen binding (purple), and response to type I interferon (pink). (**B**) Network of 111 downregulated proteins in iFECD *TCF4*ΔCTG cells compared to iFECD cells. Colored edges represent functional associations related to extracellular structure organization (blue), muscle contraction (orange), cell-substrate adhesion (green), actin cytoskeleton (purple), and extracellular matrix organization (pink).

Protein–protein interaction (PPI) network analysis using GeneMANIA revealed distinct functional clusters among the differentially expressed proteins. The upregulated protein network (Fig. 6A) displayed significant enrichment in interferon-related pathways, including type I interferon signaling, interferon-gamma-mediated signaling, and peptide antigen binding. In contrast, the downregulated protein network (Fig. 6B) showed extensive interactions related to ECM organization, muscle contraction, cell-substrate adhesion, and actin cytoskeleton.

Functional analysis of downregulated proteins (Table 3) confirmed significant involvement in ECM organization (FDR = 3.30 × 10^− 3^, 11 genes), extracellular structure organization (FDR = 3.30 × 10^− 3^, 11 genes), and multiple cell adhesion-related pathways. Conversely, upregulated proteins (Supplementary Table 1) were primarily associated with interferon response pathways, including response to type I interferon (FDR = 1.67 × 10^− 8^, 10 genes) and interferon-gamma-mediated signaling (FDR = 3.38 × 10^− 7^, 9 genes), as well as antigen processing and presentation pathways.

**Table 3:** Functional enrichment analysis of downregulated proteins by GeneMANIA.

Function	False discovery rate	Genes in network
Extracellular matrix organization	3.30 × 10^− 3^	11
Extracellular structure organization	3.30 × 10^− 3^	11
Muscle contraction	3.99 × 10^− 3^	9
Cell-substrate adhesion	5.05 × 10^− 3^	8
Actin cytoskeleton	5.05 × 10^− 3^	10
Proteinaceous extracellular matrix	5.05 × 10^− 3^	7
Muscle system process	5.05 × 10^− 3^	9
Blood vessel development	1.74 × 10^− 2^	8
Extracellular matrix	2.68 × 10^− 2^	8
Cell-matrix adhesion	3.20 × 10^− 2^	6
S100 protein binding	3.80 × 10^− 2^	3
Extracellular matrix part	4.04 × 10^− 2^	5
Blood vessel morphogenesis	4.29 × 10^− 2^	7
Angiogenesis	4.40 × 10^− 2^	8
Sulfur compound catabolic process	4.49 × 10^− 2^	4
Striated muscle thin filament	4.49 × 10^− 2^	3
Substrate adhesion-dependent cell spreading	6.72 × 10^− 2^	4
Structural constituent of muscle	6.80 × 10^− 2^	4
Protein oligomerization	6.80 × 10^− 2^	7
Myofilament	6.96 × 10^− 2^	3

## Discussion

In this study, we demonstrated that deletion of the *TCF4* CTG repeat expansion in FECD cells induces substantial proteomic remodeling that affects two major functional groups. Downregulated proteins are predominantly associated with ECM organization and cell adhesion pathways, which are functions directly related to guttae formation and corneal endothelial barrier integrity. Conversely, the upregulated proteins showed significant enrichment in interferon signaling pathways, suggesting unexpected immunomodulatory effects of the trinucleotide repeat expansion. These distinct proteomic signatures provide novel insights into how the *TCF4* expansion disrupts multiple cellular pathways to induce FECD pathogenesis.

Our finding of significant dysregulation of ECM-related proteins in our model system corroborates earlier reports of abnormal ECM composition in Descemet’s membranes from FECD patients^22–24^. This ECM dysregulation likely contributes directly to guttae formation—the clinical hallmark of FECD—and may further impact corneal endothelial cell function through ECM–cell signaling interactions, creating a pathological feedback loop central to disease progression^3,25^. Importantly, our isogenic cell model demonstrates that the removal of the *TCF4* CTG repeat expansion attenuates these ECM alterations, thereby establishing a causal link between the trinucleotide expansion and pathogenic ECM remodeling.

Emerging evidence indicates that epithelial–mesenchymal transition (EMT) programs contribute substantially to FECD pathogenesis, extending beyond simple ECM accumulation. Corneal endothelial cells from FECD patients exhibit increased expression of canonical EMT drivers such as ZEB1 and Snail1^26^, together with junctional disorganization and TGF-β-dependent upregulation of ECM proteins^26^. These findings suggest that chronic TGF-β exposure, oxidative stress, and related stress pathways may converge to induce a partial EMT state that links cellular injury with fibrotic matrix remodeling. The importance of EMT-related mechanisms in FECD is further supported by recent reviews highlighting multiple pathogenic pathways—including TCF4 repeat expansion, ER and oxidative stress, mitochondrial dysfunction, RNA toxicity, and EMT signaling—in the diseased endothelium^27^. Collectively, these observations position EMT as an important amplifier of ECM dysregulation in FECD and provide essential context for interpreting our transcriptomic findings, particularly with respect to CTG-sensitive genes implicated in EMT regulation.

Within this mechanistic framework, several CTG-sensitive genes identified in our dataset have recognized roles in EMT pathways across diverse biological systems. Neuropilin-1 interacts with fibronectin-1 to enhance EMT-associated malignant progression and also promotes EMT through NF-κB activation^28,29^. Tissue transglutaminase 2 (TGM2) likewise induces EMT by modulating MMP7 expression via MEK/ERK signaling, leading to increased cellular migration and invasion^30^. EMT activation through c-Jun stabilization following loss of 14-3-3σ further illustrates the diverse stress-responsive mechanisms that can reinforce mesenchymal transition^31^. Although these mechanisms have been described in non-ocular systems, they support the broader concept that CTG-dependent gene dysregulation in FECD may engage conserved EMT pathways. In this context, altered expression of these EMT-associated genes provides a biologically plausible link between TCF4 repeat expansion, ECM remodeling, and mesenchymal transition, yielding additional insight into FECD pathogenesis and highlighting potential therapeutic nodes within EMT signaling.

Interestingly, our observation of the upregulation of interferon-related pathways following CTG repeat deletion may point to previously unexplored aspects of FECD pathophysiology. While the role of inflammatory processes in FECD remains as yet unelucidated, our findings align with stress response signatures previously reported in FECD samples^32^. In particular, several of the most DEPs provide further mechanistic insight. Neuropilin-1, which showed the greatest upregulation, is known to mediate extracellular matrix remodeling and angiogenic signaling^33,34^, suggesting that its elevation may reflect a compensatory response to endothelial stress. Likewise, Cip1-interacting zinc finger protein and protein-glutamine gamma-glutamyltransferase 2, both markedly increased in the CTG-deleted cells, are associated with cell cycle regulation^35,36^ and extracellular matrix crosslinking^37,38^, respectively, which may contribute to altered ECM homeostasis. Conversely, strongly downregulated proteins such as alpha-crystallin B chain and 14-3-3 protein sigma, both of which play key cytoprotective and anti-apoptotic roles^39–42^, may underlie increased endothelial susceptibility in FECD. This potential connection between *TCF4* repeat expansion and immune-related pathways warrants further investigation as a possible contributory factor to the complex pathogenesis of FECD. Our data primarily support the central role of aberrant ECM deposition in disease progression, while suggesting additional cellular mechanisms that might be involved in the multifaceted pathology of this corneal dystrophy.

Our study also highlights the broader impact of the CTG repeat expansion on the corneal endothelial proteome and provides important connections to the established transcriptomic changes in FECD. Wieben et al. demonstrated that the *TCF4* CTG repeat expansion leads to widespread RNA splicing abnormalities in FECD, with particular effects on genes involved in cellular stress responses and ECM regulation^16,19^. Our proteomic data extend these findings by confirming that mRNA-level disturbances do indeed translate into functional protein changes. This transcriptome–proteome correlation strengthens the RNA toxicity hypothesis, whereby sequestration of splicing factors by expanded CUG-containing transcripts leads to downstream alterations in protein expression and cellular function^14,15^. Notably, our findings parallel observations made in studies on myotonic dystrophy type 1 (DM1), another trinucleotide repeat expansion disorder where CUG repeats cause similar splicing defects and proteomic alterations^43–46^. These mechanistic similarities^14,15^ suggest the potential for common therapeutic approaches that target RNA toxicity or its downstream effects to treat trinucleotide repeat disorders.

Our findings provide mechanistic insights with therapeutic implications. We previously demonstrated that the excessive ECM production in FECD induces endoplasmic reticulum (ER) stress through unfolded protein accumulation and contributes to corneal endothelial dysfunction^47,48^. The normalization of ECM protein expression upon CTG repeat deletion, as observed in our current study, supports a model in which the trinucleotide expansion drives both the pathological ECM deposition and the cellular stress pathways. Our results provide additional evidence supporting the therapeutic potential of antisense oligonucleotides and CRISPR-based approaches to target the *TCF4* repeat expansion^49–51^ as they suggest that these strategies could simultaneously address both guttae formation and endothelial cell dysfunction in FECD.

A significant limitation of our study is the use of immortalized FECD cells, as these cells may not fully recapitulate the in vivo environment of primary corneal endothelial cells. Because cell immortalization artifacts and in vitro culture conditions could influence protein expression patterns, validation in patient-derived tissues or in vivo models is needed. Additionally, despite our comprehensive LC-MS/MS approach, low-abundance proteins may have been underrepresented in our dataset. Future studies employing single-cell proteomics or targeted assays would help refine our understanding of the molecular pathogenesis of FECD and potentially reveal additional disease mechanisms not captured in our current analysis.

In conclusion, our study demonstrates that the *TCF4* CTG repeat expansion significantly impacts the corneal endothelial proteome, primarily through dysregulation of ECM-related pathways. These findings reinforce the central role of trinucleotide repeat expansion in FECD pathogenesis while identifying potential therapeutic targets. Future research integrating multi-omics approaches in patient-derived samples will be essential to validate these present findings and develop clinically effective treatments for FECD.

## Methods

### Ethics statement

This research was conducted in accordance with the ethical principles outlined in the Declaration of Helsinki. The study protocol received approval from the Ethics Committees of both Friedrich-Alexander University Erlangen-Nürnberg (reference: 140_20 B) and Doshisha University (reference: 20009). Patients diagnosed with FECD provided written informed consent prior to participation. Tissue specimens consisting of Descemet’s membranes with attached corneal endothelial cells were collected during routine DMEK procedures performed at Friedrich-Alexander University Erlangen-Nürnberg.

### CRISPR/Cas9-mediated deletion of *TCF4* CTG repeat expansion

We investigated the functional role of CTG repeat expansion using CRISPR/Cas9 genome editing to generate *TCF4* CTG repeat knockout cells (designated iFECD *TCF4*ΔCTG) from our established immortalized FECD cell line^52,53^. Guide RNA sequences targeting regions flanking the CTG repeat were designed using computational tools. The following oligonucleotide pairs were synthesized for gRNA construction: gRNA-1 (5′-CACCGGCCCCACTTGGAAGGCGGTT-3′ and 5′-AAACAACCGCCTTCCAAGTGGGGCC-3′) and gRNA-2 (5’-CACCGCATTTATTTCGACCCTAAT-3’ and 5’-AAACATTAGGGTCGAAATAAATGC-3’), targeting the human *TCF4* gene (Gene ID: 6925). To address potential off-target effects of the CRISPR-Cas9 system, we computationally predicted potential off-target sites for both gRNA1 and gRNA2 using the Cas-Offinder tool^54^ with parameters allowing for up to 3 mismatches and 1 bulge (DNA or RNA). A comprehensive list of predicted off-target sites, their genomic locations, and associated genes is provided in Supplementary Data 1.

After annealing complementary oligonucleotides, the resulting double-stranded fragments were inserted into the LentiCrispr v2 vector (Addgene #52961). Successful cloning was verified through Sanger sequencing. For lentiviral production, 293T cells were co-transfected with the gRNA-containing vectors along with packaging plasmids psPAX2 (Addgene #12260) and pCMV-VSV-G (Addgene #8454) using Lipofectamine™ 3000 in OptiMEM-I. Viral particles were harvested after 48 h, filtered, and concentrated using Lenti-X™ Concentrator according to the manufacturer’s recommendations. Target iFECD cells at approximately 70% confluence in 6-well plates were transduced with 100 µL of concentrated viral preparation supplemented with polybrene (5 µg/mL). Following transduction, the cells underwent puromycin selection (1 µg/mL) for 5 days. The surviving cells were then diluted and distributed into 96-well plates to enable single-cell colony formation. After 17 days of growth, the well-isolated colonies were expanded for further characterization and experimentation.

To control for potential nonspecific effects of lentiviral transduction and Cas9 expression, a mock control was prepared by transducing iFECD cells with an empty LentiCrispr v2 vector lacking the gRNA insert. These mock-transduced cells were processed in parallel with the experimental groups and used as controls in subsequent analyses.

### PCR analysis of the *TCF4* CTG repeat status

Genomic DNA was extracted from the cultured cells following a standardized protocol. Briefly, cells were washed twice with Dulbecco’s modified Eagle’s medium (DMEM), enzymatically dissociated using trypsin for 3 min, and pelleted by centrifugation. The cell pellets were resuspended in phosphate buffered saline (PBS) and processed using the MonoFas^®^ gDNA Cultured Cells Extraction Kit VI (ANIMOS Inc, Saitama, Japan) according to the manufacturer’s instructions. DNA concentration and purity were assessed spectrophotometrically using a NanoDrop™ 2000 instrument (Thermo Fisher Scientific).

The CTG repeat region in the *TCF4* gene was evaluated by performing PCR amplification using custom-designed primers flanking the trinucleotide repeat sequence. The primer pair consisted of a forward primer (5’-ACTTGGTCCTTCTCCATCCCTTTGCTT-3’) and a reverse primer (5′-CCTTCTCCTCCTCCTCCTCCTCTT-3′). Amplification reactions were conducted using GoTaq^®^ qPCR Master Mix (Promega Inc., Madison, WI) with thermal cycling parameters as follows: initial denaturation at 95 °C for 30 s, followed by 35 cycles of denaturation (95 °C, 30 s), annealing (55 °C, 30 s), and extension (72 °C, 30 s). The resulting PCR products were resolved by electrophoresis on 1.5% agarose gels containing ethidium bromide for DNA visualization. Gels were documented using an Amersham Imager 600 system (GE HealthCare, Chicago, IL) under ultraviolet illumination, allowing a comparison of amplicon sizes between the control and genome-edited cell lines.

### Analysis of *TCF4* expansion status by triplet repeat primed PCR

We precisely characterized the trinucleotide repeat configuration in *TCF4* by implementing triplet repeat primed PCR (TP-PCR). Reaction mixtures were prepared containing 20 ng genomic DNA template, a fluorescently labeled forward primer (5′-AATCCAAACCGCCTTCCAAGT-3′, 10 µmol/L), a conventional reverse primer (5′-TACGCATCCCAGTTTGAGACG-3′, 10 µmol/L), and a specialized companion reverse primer containing (CAG) repeat motifs (5′-TACGCATCCCAGTTTGAGACGCAGCAGCAGCAGCAG-3′, 1 µmol/L). Additional components included dNTPs (2 mmol/L), 2× PCR buffer for KOD Fx Neo (10 µmol/L), and KOD Fx Neo high-fidelity polymerase (1.0 unit/µL) from Toyobo Co., Ltd. (Osaka, Japan). The amplification was performed using a T3000 thermocycler (Analytik Jena, Jena, Germany) with the following thermal profile: initial denaturation (98 °C, 10 s) followed by 40 cycles of denaturation (98 °C, 10 s), annealing (65 °C, 30 s), and extension (68 °C, 30 s). The resulting amplicons were subjected to fragment analysis using an ABI 3730 Genetic Analyzer (Thermo Fisher Scientific) to determine the presence and size distribution of the expanded repeats.

### Proteomic sample preparation for mass spectrometry analysis

Cultured iFECD and iFECD *TCF4*ΔCTG cells were prepared for proteomic analysis by first washing with PBS and detaching using TrypLE reagent (Thermo Fisher Scientific). After three additional PBS washes, the cell pellets were rapidly frozen in liquid nitrogen and stored at − 80 °C until processing. For protein extraction, cells were disrupted by sonication in a lysis buffer consisting of 2% sodium dodecyl sulfate (SDS) and 50 mM triethylammonium bicarbonate containing Halt™ Protease and Phosphatase Inhibitor Cocktail (Thermo Fisher Scientific). Following clarification by centrifugation, the protein concentration in the supernatant was determined using the bicinchoninic acid (BCA) protein assay.

Sample quality was assessed by separating 20 µg aliquots on 10% SDS-PAGE gels. Prior to enzymatic digestion, proteins underwent reduction with 5 mM dithiothreitol (60 °C, 1 h), followed by alkylation with 10 mM iodoacetamide (room temperature, 30 min, protected from light). Proteins were then precipitated overnight (12 h) in ice-cold acetone at 4 °C, collected by centrifugation, and reconstituted in 50 mM triethylammonium bicarbonate buffer. Enzymatic digestion was performed using sequencing-grade trypsin (Promega, Madison, WI) over a 12 h period. The resulting peptide mixtures were purified using Sep-Pak C18 Plus Light cartridges (Waters, Milford, MA). Briefly, peptides were acidified with 1% formic acid, loaded onto activated Sep-Pak columns (preconditioned with acetonitrile followed by 0.1% formic acid), washed with 0.1% formic acid, and finally eluted with 40% acetonitrile containing 0.1% formic acid. The purified peptides were subsequently dried, reconstituted in 100 mM triethylammonium bicarbonate (TEAB), and labeled using TMT10plex™ reagents (Thermo Fisher Scientific) according to the manufacturer’s protocol for quantitative proteomic analysis.

### High-pH reverse phase fractionation of peptides

TMT-labeled peptide samples were dissolved in 1 mL of high-pH reverse phase chromatography (bRPLC) buffer A (7 mM TEAB, pH 8.5) and subjected to fractionation using an XBridge BEH C18 column (Waters). Separation was achieved using an Agilent 1260 HPLC system (Agilent Technologies, Santa Clara, CA) with a gradient elution profile utilizing increasing concentrations of buffer B (7 mM TEAB, pH 8.5, containing 90% acetonitrile). The chromatographic separation was performed at a constant flow rate of 0.3 mL/min with UV detection at 280 nm. The 90 min fractionation process generated a total elution volume of 27 mL, collected as 96 individual fractions. To reduce sample complexity while maintaining separation efficiency, these fractions were strategically combined into 12 consolidated fractions, which were subsequently dried under vacuum for downstream LC-MS/MS analysis.

### LC-MS/MS peptide analysis

Peptides were reconstituted in 0.1% formic acid and analyzed using LC-MS/MS on an Orbitrap Fusion Lumos instrument (Thermo Fisher Scientific) interfaced with an Easy-nLC™ 1200 nanoflow system (Thermo Fisher Scientific). The samples were first loaded onto a nanoViper™ precolumn (100 μm × 20 mm) at a flow rate of 3 µL/min for concentration and cleanup. They were then resolved on an Acclaim RSLC 120 C18 analytical column (75 μm × 50 cm, Thermo Fisher Scientific) with a flow rate of 280 nL/min.

Peptides were resolved using a step gradient: solvent B (0.1% formic acid in 95% acetonitrile) was increased from 8% to 22% over the first 70 min, then further raised to 35% between 70 and 103 min, with a total run time of 120 min. The mass spectrometer was operated in data-dependent acquisition mode, capturing full MS scans (m/z 350–1600) in the Orbitrap at a resolution of 120,000 (at 200 m/z). For MS1, the automatic gain control (AGC) target was set to 4 × 10^5^, with a maximum ion injection time of 50 ms. MS/MS fragmentation was conducted on the most abundant precursor ions (charge states ≥ 2) using higher-energy collisional dissociation (HCD) at 34% normalized collision energy. Fragment ions were detected in the Orbitrap at a resolution of 50,000, with a maximum injection time of 100 ms. Precursors were isolated using a 1.6 m/z window, and dynamic exclusion was applied within a 3 s cycle time.

### Proteomic data analysis

Mass spectrometric data were analyzed for protein identification and quantification using the SEQUEST™ algorithm within the Proteome Discoverer software, with searches conducted against the Human RefSeq protein database. Search parameters were designed to allow up to two missed cleavage sites, with fixed modifications including cysteine carbamidomethylation and TMT 10-plex labeling (+ 229.163 Da) at peptide N-termini and lysine residues. Methionine oxidation was considered a variable modification.

Mass tolerance was set to 10 ppm for precursor ions and 0.1 Da for fragment ions. Strict filtering criteria were applied, maintaining a false discovery rate of 1% for both peptide–spectrum matches and protein identifications. Quantitative analysis was performed using Perseus software^55^ to calculate fold changes and statistical significance. Protein abundance ratios were converted to the log_2_ scale, and DEPs were defined using dual thresholds of |log_2_ fold change| ≥ 0.5 and *P*-value < 0.05. The distribution of quantitative changes was visualized by generating volcano plots using the ggplot2 package in R, with upregulated proteins in iFECD *TCF4*ΔCTG compared to iFECD highlighted in red and downregulated proteins in blue.

Hierarchical clustering analysis was conducted using the heatmap.2 function from the gplots R package. For this analysis, the protein expression values were normalized to Z-scores (ranging from − 2 to + 2) to facilitate comparison across samples with different abundance scales. In the resulting heatmap visualization, red coloration indicated relatively high expression levels, while blue represented relatively low expression levels, enabling pattern recognition across experimental conditions.

### Western blotting

For total protein extraction, iFECD and iFECD *TCF4*ΔCTG cells were washed with ice-cold PBS and subsequently lysed in a radioimmunoprecipitation assay (RIPA) buffer. The buffer was supplemented with a protease inhibitor cocktail (Roche Applied Science, Penzberg, Germany) and a phosphatase inhibitor cocktail 2 (MilliporeSigma, Burlington, MA). The cell lysates were then clarified by centrifugation at 800 × g for 10 min. The total protein concentration in the resulting supernatants was quantified using the BCA Protein Assay Kit (Thermo Fisher Scientific).

For western blot analysis, proteins were resolved by SDS-PAGE and transferred to a PVDF membrane. The membrane was blocked for 1 h at room temperature with 3% non-fat dry milk. Subsequently, the membrane was probed overnight at 4 °C with the following primary antibodies: Neuropilin 1 (1:3000, 60067-1-lg, Proteintech Group, Inc., Rosemont, IL), CIZ1 (1:3000, 31683-1-AP, Proteintech Group, Inc), TGM2 (1:1000, PAB830Hu01, Cloud-Clone Corp, Katy, TX), PPP1R14C (1:1000, CSB-PA003096, CUSABIO Technology LLC, Houston, TX), Alpha B Crystallin (1:5000, 15808-1-AP, Proteintech Group, Inc), 14-3-3 sigma (1:5000, 66251-1-Ig, Proteintech Group, Inc), and GAPDH (1:3000, M171-3, Medical & Biological Laboratories Co., Ltd., Tokyo, Japan). After washing, the blots were incubated with horseradish peroxidase-conjugated secondary antibodies (1:5000; GE Healthcare, Chicago, IL). Immunoreactive bands were then detected using the ECL Advanced Western Blotting Detection Kit (Nacalai Tesque) via a luminal-based enhanced chemiluminescence system. Band intensities from Western blots were quantified using ImageJ software (version 1.54f).

#### Bioinformatic enrichment analysis

For a comprehensive interpretation of proteomic data, we conducted functional enrichment analyses using multiple complementary approaches. Gene Ontology (GO)^56^ classification was performed using the ClusterProfiler package (version 4.2.2) in R, with a significance threshold set at *P*-value < 0.05. The most informative GO terms (top 12) across the biological process, cellular component, and molecular function categories were selected for visualization using the ggplot2 package (version 3.3.6).

To gain insights into biological pathways affected by differential protein expression, we performed pathway enrichment analysis using Reactome^57^ databases^58,59^. Reactome pathway analysis utilized the ReactomePA package (version 1.38.0) in conjunction with ggplot2 for visualization. Significantly enriched pathways (*P*-value < 0.05) were ranked by gene ratio and displayed with a color gradient representing a significance level (*P*-values) ranging from blue (less significant) to red (highly significant).

Protein–protein interaction networks were constructed using GeneMANIA (http://genemania.org/), an online tool that integrates multiple interaction datasets, including physical interactions, genetic interactions, pathway associations, co-expression patterns, and protein domain similarities. This analysis helped identify functional modules and potential regulatory relationships among the DEPs.

### Statistical analysis

Statistical comparisons between the two groups were performed using Welch’s t-test via R software (version 4.4.1; R Core Team)^60^. Data are presented as mean ± standard error of the mean (SEM). *P*-value < 0.05 was considered statistically significant.

## Supplementary Information

Below is the link to the electronic supplementary material.

Supplementary Material 1

Supplementary Material 2

Supplementary Material 3

Supplementary Material 4

Supplementary Material 5

## Data Availability

The mass spectrometry proteomics data have been deposited to the ProteomeXchange Consortium via the PRIDE^61^ partner repository with the dataset identifier PXD075094. All proteomic data generated and analyzed during this study are provided as Supplementary Data 2.
