# Supplementary material for: TCF4 trinucleotide repeat expansion drives distinct proteomic signatures in Fuchs endothelial corneal dystrophy
Source: Sci Rep. 2026 Mar 21;16:14446. doi: 10.1038/s41598-026-43789-x (PMC13149823; doi:10.1038/s41598-026-43789-x)
Supplement: Supplementary file 1 — Supplementary Material 1 [file 41598_2026_43789_MOESM1_ESM.pdf]

Supplementary table 1. Functions of upregulated proteins analyzed by GeneMANIA

Function	FDR	Genes in network
Response to type I interferon	1.67×10^{-8}	10
Cellular response to type I interferon	1.67×10^{-8}	10
Type I interferon signaling pathway	1.67×10^{-8}	10
Interferon-gamma-mediated signaling pathway	3.38×10^{-7}	9
Peptide antigen binding	7.23×10^{-7}	6
Cellular response to interferon-gamma	1.53×10^{-6}	9
Phagocytic vesicle membrane	2.04×10^{-6}	6
Response to interferon-gamma	5.03×10^{-6}	9
Antigen processing and presentation of exogenous peptide antigen via MHC class I, TAP-dependent	5.88×10^{-6}	8
Antigen processing and presentation of exogenous peptide antigen via MHC class I	7.27×10^{-6}	8
ER to Golgi transport vesicle membrane	1.24×10^{-5}	6
ER to Golgi transport vesicle	2.28×10^{-5}	6
Early endosome membrane	2.28×10^{-5}	6
Antigen processing and presentation of peptide antigen via MHC class I	2.31×10^{-5}	8
Antigen processing and presentation of endogenous antigen	1.34×10^{-4}	4
Transport vesicle membrane	1.51×10^{-4}	6
Lumenal side of membrane	2.13×10^{-4}	5
Integral component of lumenal side of endoplasmic reticulum membrane	2.13×10^{-4}	5
Lumenal side of endoplasmic reticulum membrane	2.13×10^{-4}	5
Antigen binding	3.57×10^{-4}	6
Phagocytic vesicle	3.57×10^{-4}	6
Antigen processing and presentation of exogenous peptide antigen	1.20×10^{-3}	8
Antigen processing and presentation of exogenous antigen	1.44×10^{-3}	8
Antigen processing and presentation of peptide antigen	1.86×10^{-3}	8
Transport vesicle	4.18×10^{-3}	6
Response to virus	4.71×10^{-3}	8
Endocytic vesicle membrane	4.71×10^{-3}	6
Integral component of endoplasmic reticulum membrane	4.77×10^{-3}	6
Intrinsic component of endoplasmic reticulum membrane	4.89×10^{-3}	6
Coated vesicle membrane	5.64×10^{-3}	6
Peptide binding	5.93×10^{-3}	6
Antigen processing and presentation	5.93×10^{-3}	8
