# Supplementary material for: TCF4 trinucleotide repeat expansion drives distinct proteomic signatures in Fuchs endothelial corneal dystrophy
Source: Sci Rep. 2026 Mar 21;16:14446. doi: 10.1038/s41598-026-43789-x (PMC13149823; doi:10.1038/s41598-026-43789-x)
Supplement: Supplementary file 4 — Supplementary Material 4 [file 41598_2026_43789_MOESM4_ESM.pdf]

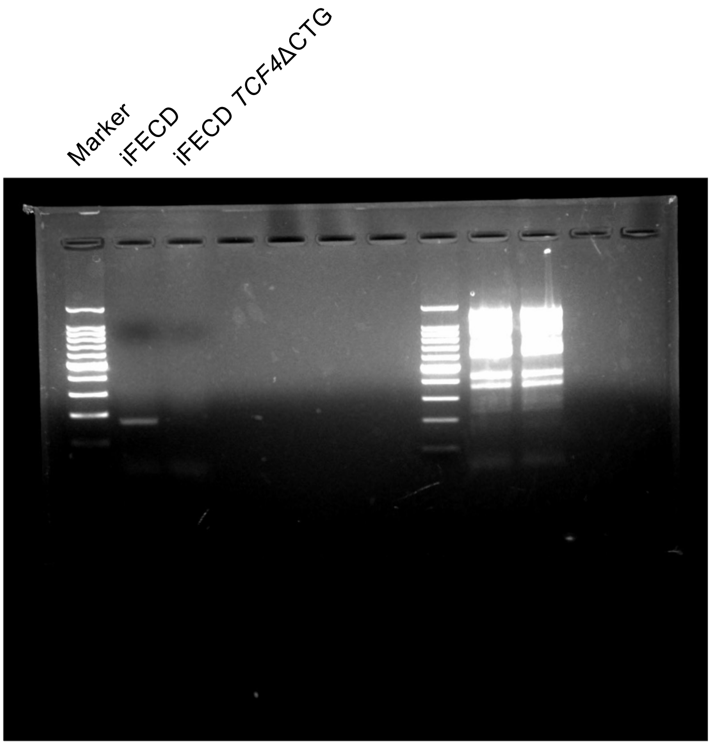

Supplementary Figure 1. Unprocessed PCR gel image

The original, unprocessed agarose gel image corresponding to the PCR experiment shown in Figure 1B. Lanes represent the DNA marker, iFECD, and iFECD *TCF4*ΔCTG samples. The three lanes on the right side contain unrelated samples that were run on the same gel. No adjustments or modifications were applied to brightness or contrast.
