# Supplementary material for: TCF4 trinucleotide repeat expansion drives distinct proteomic signatures in Fuchs endothelial corneal dystrophy
Source: Sci Rep. 2026 Mar 21;16:14446. doi: 10.1038/s41598-026-43789-x (PMC13149823; doi:10.1038/s41598-026-43789-x)
Supplement: Supplementary file 5 — Supplementary Material 5 [file 41598_2026_43789_MOESM5_ESM.pdf]

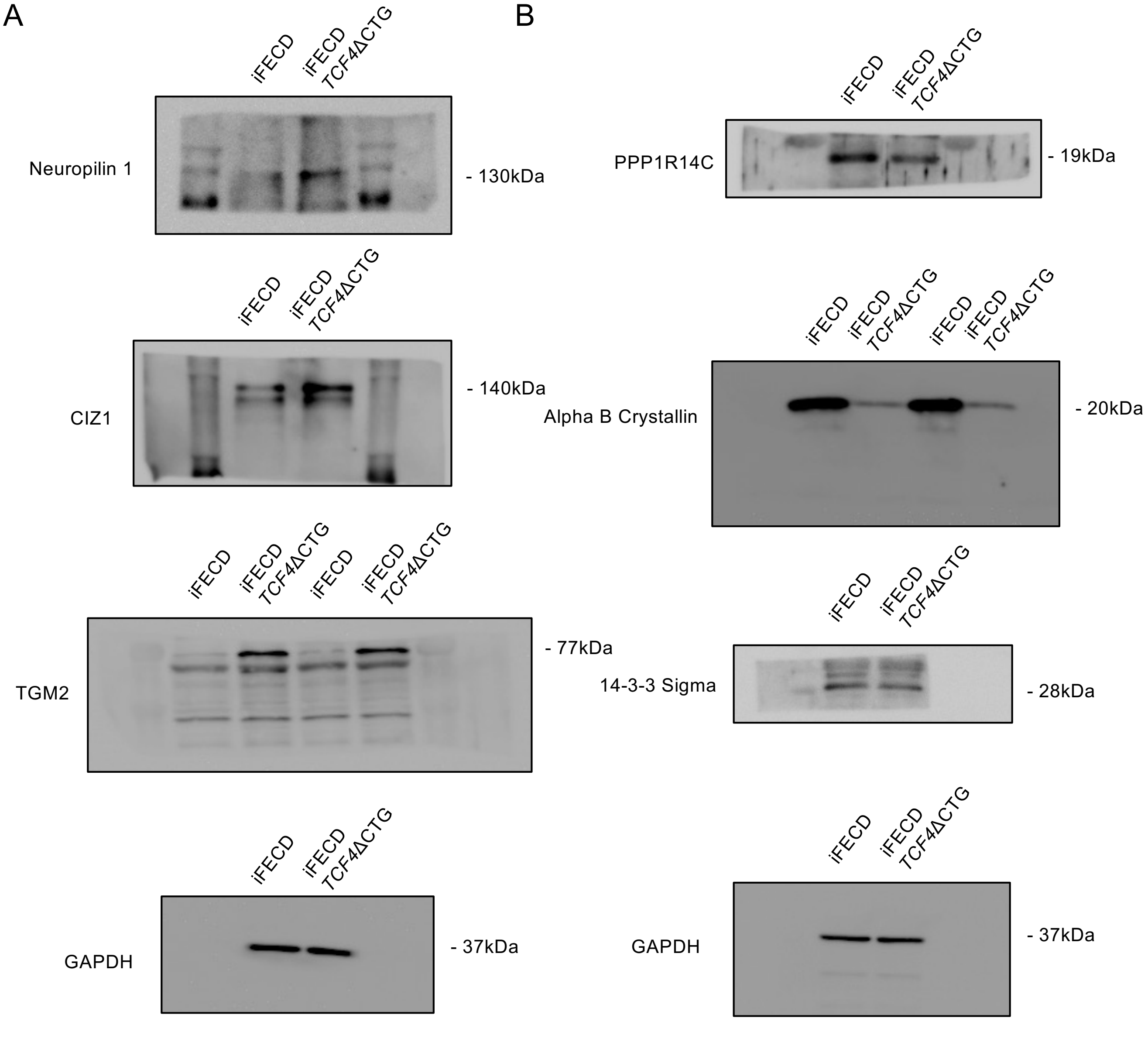

Supplementary Figure 2. Unprocessed membrane images of western blotting

Unprocessed full-length membrane images of western blotting analyses. **(A)** Original blots for Neuropilin 1, CIZ1, and TGM2. **(B)** Original blots for PPP1R14C, Alpha B Crystallin, and 14-3-3 Sigma. GAPDH is included in both panels as an internal loading control. All images are presented in their original, unprocessed versions to comply with transparency requirements. The positions of molecular weight markers (kDa) are indicated on the right of each blot. Note the presence of non-specific bands in several blots, particularly for TGM2 and 14-3-3 sigma.
